# Double Stimulation in a Spiking Neural Network Model of the Midbrain Superior Colliculus

**DOI:** 10.3389/fams.2018.00047

**Published:** 2018-10-09

**Authors:** Bahadir Kasap, A. John van Opstal

**Affiliations:** Department of Biophysics, Donders Institute for Brain, Cognition and Behavior, Radboud University, Nijmegen, Netherlands

**Keywords:** saccades, motor map, spatial-temporal transformation, electrical stimulation, population coding, vector averaging

## Abstract

The midbrain superior colliculus (SC) is a crucial sensorimotor interface in the generation of rapid saccadic gaze shifts. For every saccade it recruits a large population of cells in its vectorial motor map. Supra-threshold electrical microstimulation in the SC reveals that the stimulated site produces the saccade vector specified by the motor map. Electrically evoked saccades (E-saccades) have kinematic properties that strongly resemble natural, visual-evoked saccades (V-saccades), with little influence of the stimulation parameters. Moreover, synchronous stimulation at two sites yields eye movements that resemble a weighted vector average of the individual stimulation effects. Single-unit recordings have indicated that the SC population acts as a vectorial pulse generator by specifying the instantaneous gaze-kinematics through dynamic summation of the movement effects of all SC spike trains. But how to reconcile the a-specific stimulation pulses with these intricate saccade properties? We recently developed a spiking neural network model of the SC, in which microstimulation initially activates a relatively small set of (~50) neurons around the electrode tip, which subsequently sets up a large population response (~5,000 neurons) through lateral synaptic interactions. Single-site microstimulation in this network thus produces the saccade properties and firing rate profiles as seen in single-unit recording experiments. We here show that this mechanism also accounts for many results of simultaneous double stimulation at different SC sites. The resulting E-saccade trajectories resemble a weighted average of the single-site effects, in which stimulus current strength of the electrode pulses serve as weighting factors. We discuss under which conditions the network produces effects that deviate from experimental results.

## Introduction

### Superior Colliculus

Because high spatial resolution is limited to the central fovea, the primate visual system needs to explore the environment through rapid and precise saccadic eye movements. Normal (human and monkey) saccades display stereotyped “main sequence” characteristics, described by linear amplitude-duration and nonlinear, saturating, amplitude-peak eye velocity relationships [1]. In addition, the horizontal and vertical velocity profiles of oblique saccades are tightly coupled, such that they are scaled versions of each other throughout the saccade, and saccade trajectories are approximately straight in all directions [2]. These properties imply that the saccadic system contains a nonlinear control stage [2–4].

Previously, these main-sequence properties had been assumed to arise at the brainstem level, possibly because of saturation of the brainstem saccadic burst neurons [3].

Recent hypotheses have suggested, however, that the saccade nonlinearity reflects a speed-accuracy trade-off, which optimally deals with spatial uncertainty in the retinal periphery and internal noise in sensorimotor pathways [5–8]. We have hypothesized that the midbrain superior colliculus (SC) would be in an excellent position to implement such a strategy [8].

**Figure 1:**
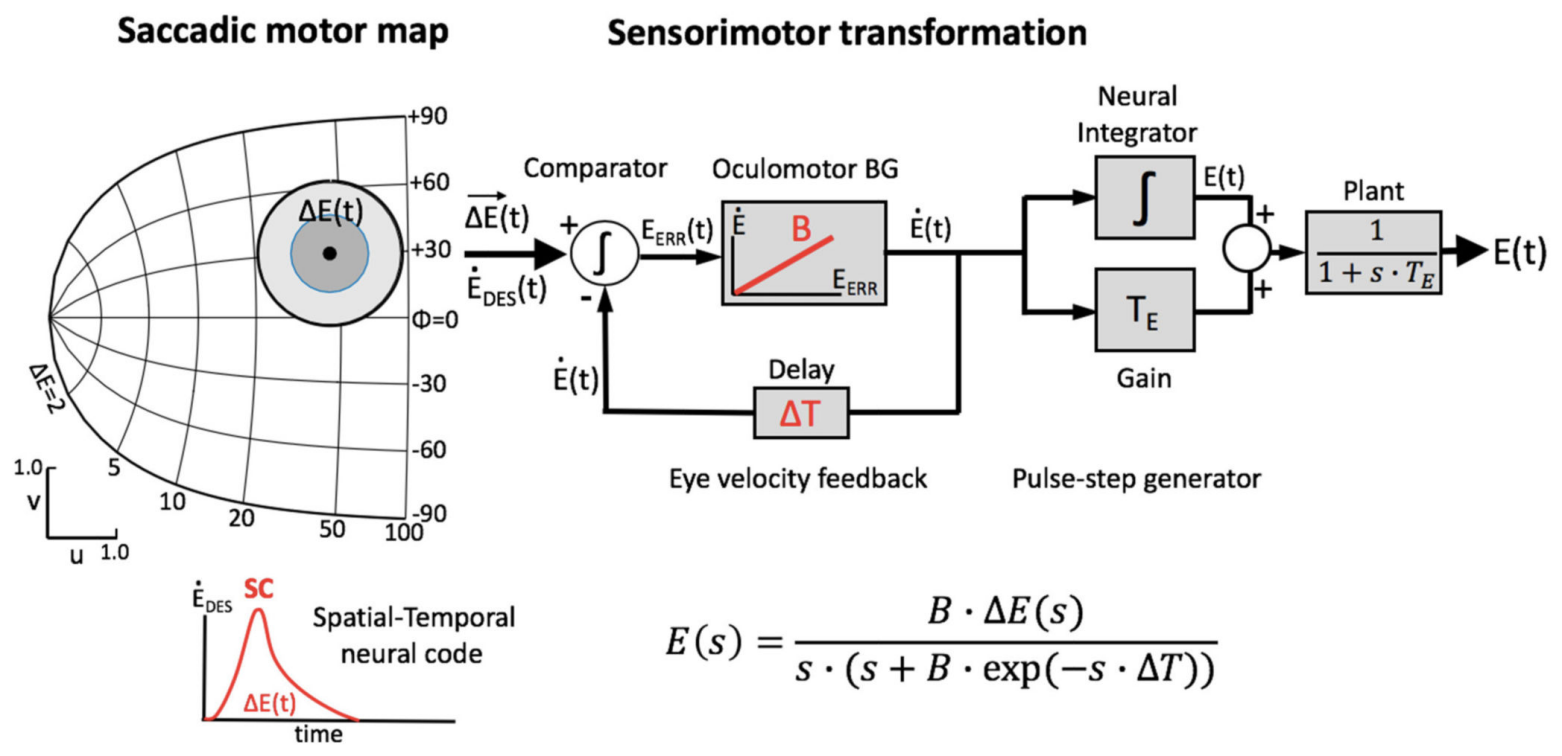
Simplified schematic representation of our model of the saccadic system (after [13]). The SC motor map (Equation 1a) encodes the upcoming saccade by recruiting a population of cells at the appropriate location (Equation 2), and setting up a firing rate profile (see inset) that specifies the desired trajectory and kinematics of the eye. At the comparator, this dynamic signal is continuously compared with the ongoing true eye velocity (delay, ΔT), and their integrated difference represents the dynamic motor error, E_err_(t). The latter drives the brainstem burst generator, which is represented by a simple linear gain (B). The BG provides the velocity pulse for the pulse-step generator, which drives the oculomotor plant. Note that the total model is entirely linear, and has only two free parameters (B and ΔT). The equation provides the Laplace transfer function between the SC output, ΔE(s), and the eye movement response, E(s), with s the complex Laplace variable. Note that the transfer is independent of the plant’s time constant. Yet, when driven by measured SC spike trains, the model produces the full nonlinear kinematics of saccades. As a logical result of this observation, the nonlinearity has to reside in the encoding of the SC burst.

The neural circuitry underlying saccade planning, selection, and execution extends from the cerebral cortex to the cerebellum, and the pons in the brainstem. The midbrain SC is the final common terminal for all cortical and subcortical outputs, and it is known to specify the vectorial eye-displacement command for the brainstem oculomotor circuitry [9–11]. The SC contains an eye-centered topographic map of visuomotor space, in which the saccade amplitude is mapped logarithmically along the rostral-caudal axis (*u*, in mm) and saccade direction roughly linearly along the medial-lateral direction (*v*, in mm; [9]). The afferent mapping (Equation 1a) and its efferent inverse (Equation 1b) are well described by Ottes et al. [12]:

(1a) $$\begin{array}{l} u=B_{u}\;\text{ln}\left(\frac{\sqrt{{\left(x+A\right)}^{2}+y^{2}}}{A}\right)\\ v=\;B_{v}\;\text{atan}\left(\frac{y}{x+A}\right) \end{array}\}$$

(1b) $$\Leftrightarrow \left\{ \begin{array}{l} x=A\cdot \left(\text{exp}\left(\frac{u}{B_{u}}\right)\text{cos}\left(\frac{v}{B_{v}}\right)-1\right)\\ \;y=A\cdot \text{exp}\left(\frac{u}{B_{u}}\right)\text{sin}\left(\frac{v}{B_{v}}\right) \end{array}\right.$$

with typical parameter values for the monkey SC given as B_u_≈1.4 mm, B_v_≈1.8 mm/rad, and A≈3 deg; see Figure 1). Each saccade is associated with a translation-invariant Gaussian-shaped population within this map, the center of which corresponds (through Equation 1a) to the saccade vector, (*x*_0_*,y*_0_), and a width *σ*_pop_≈0.5 mm [12, 14, 15]. Thus, the activity of neuron *n* in the motor map is described by:

(2) $$F_{n}\left(u_{n},v_{n}\right)=F_{\text{max}}\cdot e^{-\frac{1}{2}\cdot \left(\frac{{\left(u_{0}-u_{n}\right)}^{2}+{\left(v_{0}-v_{n}\right)}^{2}}{\sigma_{pop}^{2}}\right)}$$

with *F*_max_ the peak activity of the population, quantified by the number of spikes in the saccade-related burst (e.g., Figures 1, 3A). It is generally assumed that each recruited neuron, *n*, in the population encodes a vectorial movement contribution to the saccade vector, which is determined by both its anatomical location within the motor map, *(u_n_,v_n_)*, and its activity, *F_n_* [2, 11–13, 16–18].

However, the precise mechanism by which the cells contribute to the saccade is still elusive. A major hypothesis in the literature holds that the output of the population is determined by a nonlinear center-of-gravity computation [17–21]. According to this idea, the activity in the SC motor map only specifies the saccade metrics (amplitude and direction of the saccade vector) and is unrelated to the saccade kinematics. Yet, our single-unit recordings demonstrated a strong (presumably causal) relationship between the instantaneous firing patterns in the SC and associated saccade trajectories [8, 13].

We therefore proposed and tested an extremely simple linear summation model for the recruited population that explains the encoding of spatial-temporal properties of saccade trajectories through the firing properties of SC burst cells ([8, 13]); Figure 1. According to this model, the saccade, ***S****(t)*, is generated in the following way:

(3) $$\text{S}\left(t\right)=\sum_{n=1}^{N}\sum_{k=1}^{K_{n}<t}\delta \left(t-\tau_{n,k}\right).{\text{m}}_{n}$$

with *N* the number of active cells in the population, *K_n_*<t the number of spikes in the burst of neuron *n* up to time *t*, and **m**_n_ = ζ·*(x_n_,y_n_)* the tiny site-specific spike vector emanating from the motor map for each spike from each cell. This spike vector is solely determined by the efferent mapping of SC site *(u_n_,v_n_)* (Equation 1b), where ζ is a fixed, small scaling constant determined by the cell density in the map and the population size, and *δ(t*-*τ*_*k*,*n*_*)* is the *k*’th spike fired by neuron *n* at time *τ*_k,n_.

Our linear dynamic ensemble-coding model is illustrated in Figure 1. The SC provides a feedforward motor command by the temporal integration of all spike trains of the total population. The integrated signal represents the cumulative desired displacement of the eye, whereas the population firing rate represents the desired eye velocity (inset). The SC output thus represents both a spatial (by the location of the population) and a temporal (the instantaneous firing rates) neural code of the eye movement. The SC signal is continuously compared with an efference copy of the true eye velocity (with delay, ΔT), which is generated by the brainstem saccadic burst generator (BG). Note that in our model the BG is taken as a simple no-memory linear system (gain, B). The BG output is subsequently fed through a parallel circuit, consisting of the eye-position integrator and a static gain (T_E_). These signals combine at the oculomotor neurons to produce the pulse-step innervation for the oculomotor plant. The latter is usually modeled by a simple first-order low-pass filter with time constant T_E_. We showed that this entirely linear model resulted to account for the full nonlinear kinematics of saccades. We therefore proposed that the main-sequence properties should originate at the level of the SC motor map [8, 13]. The neural mechanism underlying this property was identified as a precise tuning of the peak firing rates and burst durations in the SC as a function of their location in the map, while keeping the number of spikes in the population fixed. As a result, the instantaneous firing rates of the neurons together encode all measured properties of saccadic velocity profiles [22].

Recently, we implemented a simple spiking neural network model for the SC that can generate realistic saccades to visual targets [23]. This minimalistic (one-dimensional) model with lateral excitatory-inhibitory interactions among the SC cells accounts for most of the experimentally observed firing properties of saccade-related neurons in the motor map [8, 13], and yields saccades with normal main-sequence properties. The model takes a fixed Gaussian input from upstream sources (e.g., the cortical frontal eye fields, or FEF), and assumes precisely-tuned biophysical properties of the SC network neurons, and their interconnections.

### Microstimulation

Electrical stimulation at a particular site in the motor map produces a saccadic gaze shift with metrics that correspond well to the efferent mapping function (Equation 1b), and with normal main-sequence kinematics [9, 15, 24, 25]. These studies have also shown that the properties of electrically evoked (E-)saccades are largely invariant to a wide range of stimulation parameters, which might appear problematic for the linear ensemble-coding model of Equation 3.

Note that two factors contribute to the neural responses to electrical microstimulation: (1) direct (feedforward) current activation of cell bodies and axons by the electric field of the electrode, and (2) synaptic activation through lateral (feedback) interactions among the neurons in the motor map [26].

We recently argued that as current strength falls off rapidly with distance from the electrode tip, only a small number of SC neurons will be directly stimulated by the electrode’s electric field (e.g., [27]). Thus, the major factor determining the microstimulation effects would be synaptic transmission. Indeed, several studies have suggested the existence of a functional organization of lateral excitatory-inhibitory interactions within the SC (anatomy: [28, 29]; electrophysiology: [30–32], and pharmacology: [33]).

We thus extended our spiking model to account for single-site microstimulation results over a wide range of stimulation parameters [26]. The network was tuned such that, above a threshold, the E-saccades were insensitive to changes in the stimulation parameters. This result supports the idea that the excitatory-inhibitory interactions effectively normalize the total SC output. Under microstimulation, the network thus creates a population that is virtually identical to the one elicited by a visual stimulus. It may be expected that such intrinsic normalization could ensure a behavior that resembles (nonlinear) weighted-averaging without the need for a nonlinear, activation-dependent weighting scheme that is implemented downstream from the motor map.

### Double Stimulation

In this paper, we further explored the predictions of our model for synchronous and asynchronous electrical stimulation at two different sites. Robinson [9] and Nota and Gnadt [34] demonstrated that double stimulation in the SC produced eye movements that resemble the weighted average of the individual stimulation effects, with the stimulation current strengths and relative timings acting as weighting factors. Similar weighting effects occur when an electrical stimulus is combined with a behaviorally relevant visual stimulus [35]. Results such as these have prompted computational modelers to propose a downstream vector-averaging mechanism that acts on the SC output by explicitly calculating the center of gravity of the population (see above; [17–21]; review in [36]). The neural mechanism that would implement such a neural computation, however, remains unspecified.

**Figure 2:**
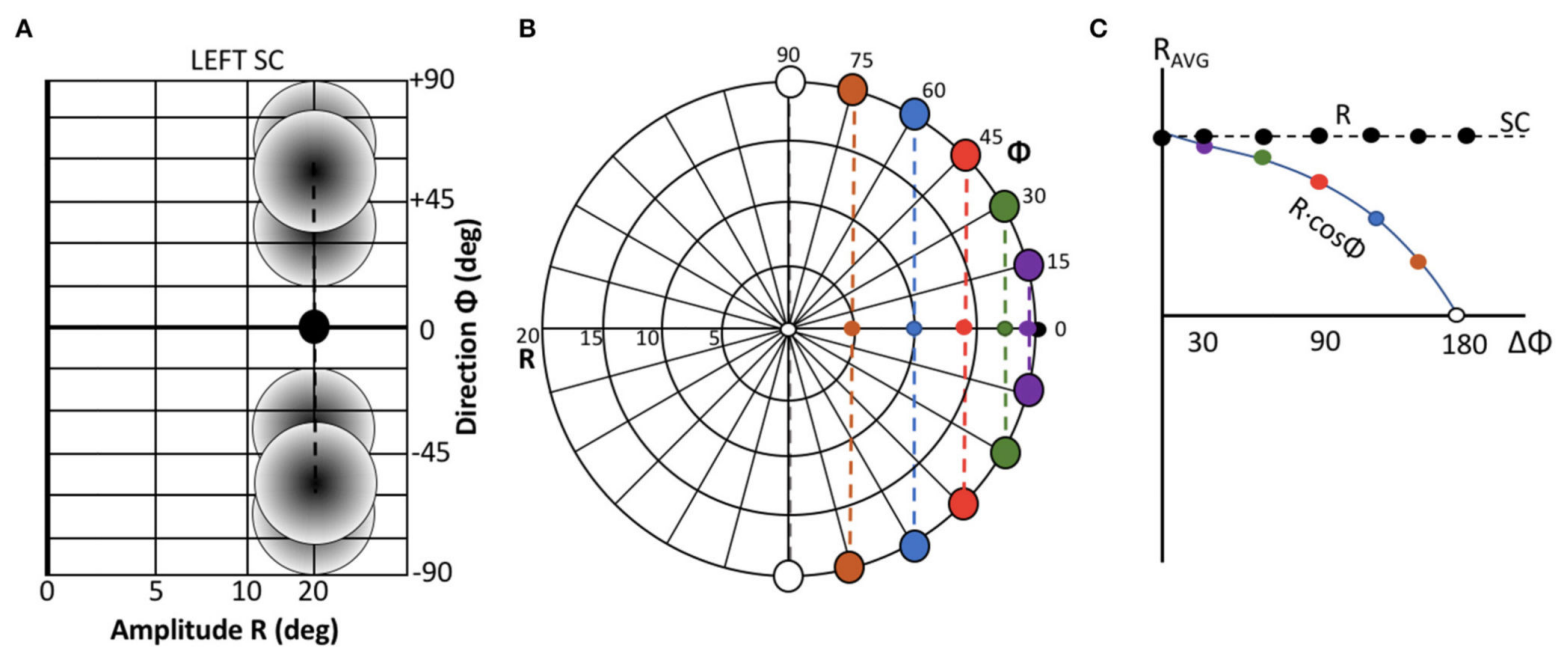
Geometrical consequences of center-of-gravity averaging at the SC level vs. downstream from the motor map. (**A**) Hypothetical double-stimulation effects for two sites at eccentricity R = 20 deg, placed symmetrically around the horizontal meridian at *Φ* = 0 deg, with angular separation of 60, 100, and 160 deg, respectively. Weighted averaging within the map (Equation 4a) would effectively lead to a horizontal movement corresponding to (R,Φ) = (20, 0) deg for all three situations (black dot). (**B**) If this process occurs downstream from the motor map, the averaged movement (Equation 4b) would be horizontal, but with an amplitude that systematically depends on the separation angle [colored dots; black dot: result of (A)]. (**C**) Predictions for the two different center-of-gravity mechanisms.

Figure 2 illustrates two extreme outcomes for mechanisms that would both calculate the center of gravity (CoG) of the effects of the total activity: averaging at the level of the motor map (Equation 4a), vs. averaging at the level of the brainstem (Equation 4b), i.e.,:

(4a) $${\vec{S}}_{\text{CoG}}^{SC}=\frac{\sum_{n=1}^{N_{\text{POP}}}F_{n}\cdot {\vec{w}}_{n}}{\sum_{n=1}^{N_{\text{POP}}}F_{n}}\;\text{with}\;{\vec{w}}_{n}=\left(u_{n},v_{n}\right)$$

(4b) $$\text{vs}.{\vec{S}}_{\text{CoG}}^{\text{DOWN}}=\frac{\sum_{n=1}^{N_{\text{POP}}}F_{n}\cdot {\vec{m}}_{n}}{\sum_{n=1}^{N_{\text{POP}}}F_{n}}\;\text{with}\;{\vec{m}}_{n}=\left(x_{n},y_{n}\right)$$

Note that in the former case (Figure 2A), the resulting saccade is horizontal with a constant amplitude of 20 deg, regardless the direction of the single-site responses. In the case of Equation (4b), however, response amplitude varies with the angle, Φ, of the single-site stimulation response as R_CoG_ = *R_SITE_* · cos Φ_*SITE*_ (Figure 2B).

In an earlier modeling study we had shown that lateral excitatory/inhibitory synaptic interactions within the SC motor map, in combination with the linear ensemble-coding scheme of Van Gisbergen et al. [14], could account for saccade-averaging effects to (synchronous) double stimulation [37, 38]. However, the model’s output of that study only focused on the saccade-vector endpoints, as it was not equipped to generate saccade trajectories and their kinematics.

Here we employ the dynamic ensemble-coding scheme of Equation (3) to our spiking collicular network to simulate two-dimensional saccade trajectories under a variety of electrical double-stimulation conditions. We show that linear dynamic ensemble-coding with lateral excitatory-inhibitory interactions in the motor map can account for most of the experimental vector-averaging results to double stimulation [9, 20, 35], without the need for additional computational nonlinearities, such as a downstream population center-of-gravity computation [20, 21, 34], or a spike-counting cut-off threshold [13, 39, 40]. The results of our model simulations suggest several interesting limiting cases to the averaging behavior, which, to our knowledge, have so far not been investigated in experimental studies. We also discuss to what extent the model’s responses deviate from experimental findings, and suggest some further refinements to the model.

## Methods

### The Log-Polar Mapping

Without loss of generality, we simplified the afferent motor map of Equation (1a) to the isotropic complex logarithmic function, by setting *B_u_* = *B_v_* = 1, and *A* = 0:

(5a) $$\begin{array}{l} u\left(R\right)=\text{ln}\left(R\right)\;\text{and}\;v\left(\phi \right)=\phi ,\;\text{with}\;R=\sqrt{x^{2}+y^{2}}\;\text{and}\\ \;\phi =\text{atan}\left(\frac{y}{x}\right) \end{array}$$

Thus, a single spike’s movement contribution to the saccade from a cell at site (*u,v*) is determined by the simplified efferent mapping relations:

(5b) $$m_{x}\left(u,v\right)=\zeta \cdot \text{exp}\;\left(u\right)\cdot \text{cos}\;\left(v\right)\;\text{and}\;m_{y}\left(u,v\right)=\zeta \cdot \text{exp}\;\left(u\right)\cdot \text{sin}\;\left(v\right)$$

We modeled the spiking neural network by a rectangular grid of 201 x 201 neurons, representing the gaze motor-map of the right hemifield with 0 < *u* < 5 mm (i.e., up to *R* = 148 deg), and $\text{-}\frac{\pi }{2}<v<\frac{\pi }{2}\;\text{mm}.$ Under single-site stimulation, the center location of the recruited population determines the direction and amplitude of the saccade, whereas the temporal activity profile encodes the eye-movement kinematics through Equation (3). As described in our previous studies [23, 26], and briefly summarized below (Equations 13 and 14), the eye-movement main-sequence kinematics result from the location-dependent biophysical properties of the neurons, and their lateral excitatory-inhibitory connectivity profiles.

### The Adex Neuron Model

We studied the dynamics of the network through simulations developed in C++/CUDA [41], by custom code that implemented dynamic parallelism on a GPU [42], developed and tested on a Tesla K40 with CUDA Toolkit 7.0, Linux Ubuntu 16.04 LTS. Simulations ran with a time resolution of 0.01 ms. Brute-force search and genetic algorithms were used for parameter identification and network tuning since there exists no analytical solutions for the system [23, 26]. Sample simulation and analysis code can be found under https://bitbucket.org/bkasap/sc_doublestimulation/.

Neurons were described by the adaptive exponential integrate- and-fire (AdEx) model [43, 44], which is a conductance-based model with an exponential membrane potential dependence. The nonlinear temporal dynamics of neuron *n* are described by two coupled differential equations that determine the two state variables: the cell’s membrane potential, *V*, and the adaptation current, *q*:

(6a) $$\begin{array}{l} C\frac{dV_{n}}{dt}=-g_{L}\left(V_{n}-E_{L}\right)+g_{L}\eta \;\text{exp}\left(\frac{V_{n}-V_{T}}{\eta }\right)\\ \;-q_{n}+I_{inp,n}\left(t\right) \end{array}$$

(6b) $$\tau_{q,n}\frac{dq_{n}}{dt}=a\;(V_{n}-E_{L})-q_{n}$$

*C* is the membrane capacitance, *g_L_* is the leak conductance, *E_L_* is the leak reversal potential, *η* is a slope factor, *V_T_* determines the neural spiking threshold, *τ*_*q*,*n*_ is the adaptation time constant, *a* is the sub-threshold adaptation constant, and *I*_*inp*, *n*_ is the cell’s total synaptic input current.

Once the membrane potential crosses *V_T_*, the exponential term in Equation (6a) starts to dominate. To limit the membrane potential, we incorporated a ceiling threshold at *V_peak_* = −30mV for spike generation. For each spiking event at time *τ*, the membrane potential is reset to its resting potential, *V_rst_*, and the adaptation current, *q_n_*, is increased by *b* to implement the spike-triggered neural adaptation:

(7) $$V_{n}\left(\tau \right)\to V_{rst}\;\text{and}\;q_{n}\left(\tau \right)\to q_{n}\left(\tau \right)+b$$

In our model, two biophysical parameters specify the firing properties of the SC neurons: the adaptation time constant, *τ*_*q*, *n*_ (taken to be location dependent; [23]), and the synaptic input current, *I*_*inp*, *n*_, which is partly determined by the intra-collicular connections (see below). In our model, both depend systematically on the rostral-causal location (*u*) of the cells within the network. The remaining parameters, *C*, *g_L_*, *E_L_*, *η*, *V_T_*, and *a*, were fixed and tuned such that the cells showed neural bursting behavior (see Table 1 for the list and values of all parameters used in the simulations, and [26], for example responses and phase plots).

### Current Spread

We applied electrical stimulation by the input current, centered around site *[u_E_,v_E_]*. We assumed an exponential spatial decay of the electric field from the tip of each stimulation electrode. For stimulation at a single site at time t_1_:

(8) $$I_{E}\left(u,v,t\right)=I_{0}\cdot \text{exp}\left(-\text{λ}\cdot \sqrt{{\left(u-u_{E}\right)}^{2}+{\left(v-v_{E}\right)}^{2}}\right)\cdot P\left(t-t_{1}\right)$$

with λ (mm^−1^) a spatial decay constant, *I*_0_ the current intensity at site (*u_E_,v_E_*) (in pA), and a rectangular stimulation pulse given by *P(t)* = 1 for 0 < *t* – *t*_1_ < *D*_S_, and 0 elsewhere. Thus, only a small set of neurons around the stimulation site will be directly activated with this input current (see [26]). In double-stimulation trials, two stimuli were applied at different sites. The total current is then given by:

(9) $$\begin{array}{l} I_{E}\left(u,v,t\right)=\sum_{n=1}^{2}I_{0,n}\cdot \text{exp}\left(-\text{λ}\cdot \sqrt{{\left(u-u_{E,n}\right)}^{2}+{\left(v-v_{E,n}\right)}^{2}}\right)\cdot \\ \;P_{n}\left(t-t_{n}\right) \end{array}$$

In these simulations, stimulus amplitudes, sites, durations, and their relative timings were systematically varied.

### Synapse Dynamics and Lateral Connections

The total input current for neuron *n* depends on the spiking activity of its surrounding neurons through conductance-based synaptic transmission, and external electric current inputs (Equations 8 or 9):

(10) $$\begin{array}{l} I_{inp,\;n}\left(t\right)=g_{n}^{exc}\left(t\right)\left(E_{e}-V_{n}\left(t\right)\right)+g_{n}^{inh}\left(t\right)\left(E_{i}-V_{n}\left(t\right)\right)\\ \;+I_{E}\left(u_{n},v_{n},t\right) \end{array}$$

where $g_{n}^{exc}$ and $g_{n}^{inh}$ are excitatory and inhibitory synaptic conductances acting upon neuron *n*, *E_e_*, and *E_i_* are excitatory and inhibitory reversal potentials, respectively. These conductances increase instantaneously for each presynaptic spike by a factor that is determined by the synaptic connection strength between neurons, and they subsequently decay over time in an exponential way:

(11a) $$\tau_{exc}\frac{dg_{n}^{exc}}{dt}=-g_{n}^{exc}+\tau_{exc}\sum_{i}^{N_{pop}}w_{i,\;n}^{exc}\sum_{s}^{N_{spks}^{i}}\delta \;(t-\tau_{i,s})$$

(11b) $$\tau_{inh}\frac{dg_{n}^{inh}}{dt}=-g_{n}^{inh}+\tau_{inh}\sum_{i}^{N_{pop}}w_{i,\;n}^{inh}\sum_{s}^{N_{spks}^{i}}\delta \;(t-\tau_{i,s})$$

with *τ_exc_* and *τ_inh_*, the excitatory and inhibitory time constants; $w_{i,\;n}^{exc}\;\text{and}\;w_{i,\;n}^{inh}$ are the intracollicular excitatory and inhibitory connection strengths between neurons *i* and *n*, respectively (Equations 12a,b) and *τ*_*i*, *s*_ are the spike timings of all presynaptic SC neurons projecting to neuron *n*.

We incorporated a Mexican hat-type lateral connection scheme [45]:

(12) $$w_{i,n}=s_{n}\cdot \left(w_{i,n}^{exc}-w_{i,n}^{inh}\right),\;\text{with}$$

(12a) $$w_{i,n}^{exc}={\bar{w}}_{exc}\;\text{exp}\left(-\frac{||u_{i}-u_{n}|{|}^{2}}{2\sigma_{exc}^{2}}\right)$$

(12b) $$w_{i,n}^{inh}={\bar{w}}_{inh}\;\text{exp}\left(-\frac{{\left\|u_{i}-u_{n}\right\|}^{2}}{2\sigma_{inh}^{2}}\right)$$

where ${\bar{w}}_{exc}\;>\;{\bar{w}}_{inh}\;\text{and}\;{\bar{\sigma }}_{inh}\;>\;{\bar{\sigma }}_{exc,}$ and *s_n_* is a location-dependent synaptic scaling parameter, which accounts for the location-dependent change in neuronal sensitivity that is related to the variation in their adaptation time constants. Note, that in our model each SC neuron exerts both excitatory and inhibitory effects on the other neurons in the map, depending on inter-neuron distance. Thus, for simplicity, the inhibitory connections were not mediated by a separate class of inhibitory interneurons.

Figure 1B exemplifies the connectivity profile for a single site.

The strong short-range excitatory and weak long-range inhibitory synapses act as a dynamic soft winner-take-all (WTA) mechanism: not just one neuron remains active, but the “winner” affects the temporal activity patterns of the other active neurons too. The central neuron thus governs the population activity, since it usually is the most active one (but note that under double-stimulation conditions this may change; see section Results). As a result, all recruited neurons exhibit similarly-shaped bursting profiles as the most active neuron, leading to spike-train synchronization within the population [8, 23, 26].

### Network Tuning

The intrinsic biophysical properties of the neurons were enforced by systematically varying the adaptation time constant, *τ*_*q*,*n*_, and the synaptic weight-scaling parameter, *s_n_*. Changes in the adaptive properties result in a varying susceptibility to synaptic input, while the synaptic scaling corrects for the total input activity. Following the brute-force genetic algorithm from our recent paper [23, 26], the optimal location-dependent [*τ*_*q*,*n*_, *s_n_*] value pairs for the neurons were fitted to ensure a systematic negative rostral-caudal gradient of the peak firing rates $\left(f_{peak}\propto \frac{1}{\sqrt{R}}\right)$ and a fixed number of spikes per neuron for its preferred saccade (*N_SPK_* = 20) under a single-site microstimulation condition with *I*_0_ = 150 pA and *D_S_* = 100ms.

In short, the algorithm optimized the network “fitness,” by incorporating the scaled contributions of the cells’ peak firing rates, their total spike counts, and an inter-cellular synchronization index within the recruited population. As a result, the adaptive time constant, *τ*_*q*, *n*_, decreased linearly from 100 to 30ms with the anatomical rostral-caudal location of the neuron, *u_n_*, according to:

(13) $$\tau_{q,n}=100-14*u_{n}\;\text{ms},\;\text{with}\;u_{n}\in \left[0,5\right]\text{mm}$$

The optimal synaptic scaling factor for the lateral excitatory/inhibitory connections (Equation 12) could be fitted by a monotonically decreasing 5th-order polynomial in *u _n_* (sin mm; [26]):

(14) $$\begin{array}{l} s\left(u_{n}\right)=0.0148+(-2.52\cdot u_{n}+1.6856\cdot u_{n}^{2}-1.49\cdot u_{n}^{3}\\ \;+\;0.4318\cdot u_{n}^{4}-0.04737\cdot u_{n}^{5})\cdot 10^{-4} \end{array}$$

Table 1 provides the model’s full parameter list.

**Figure 3:**
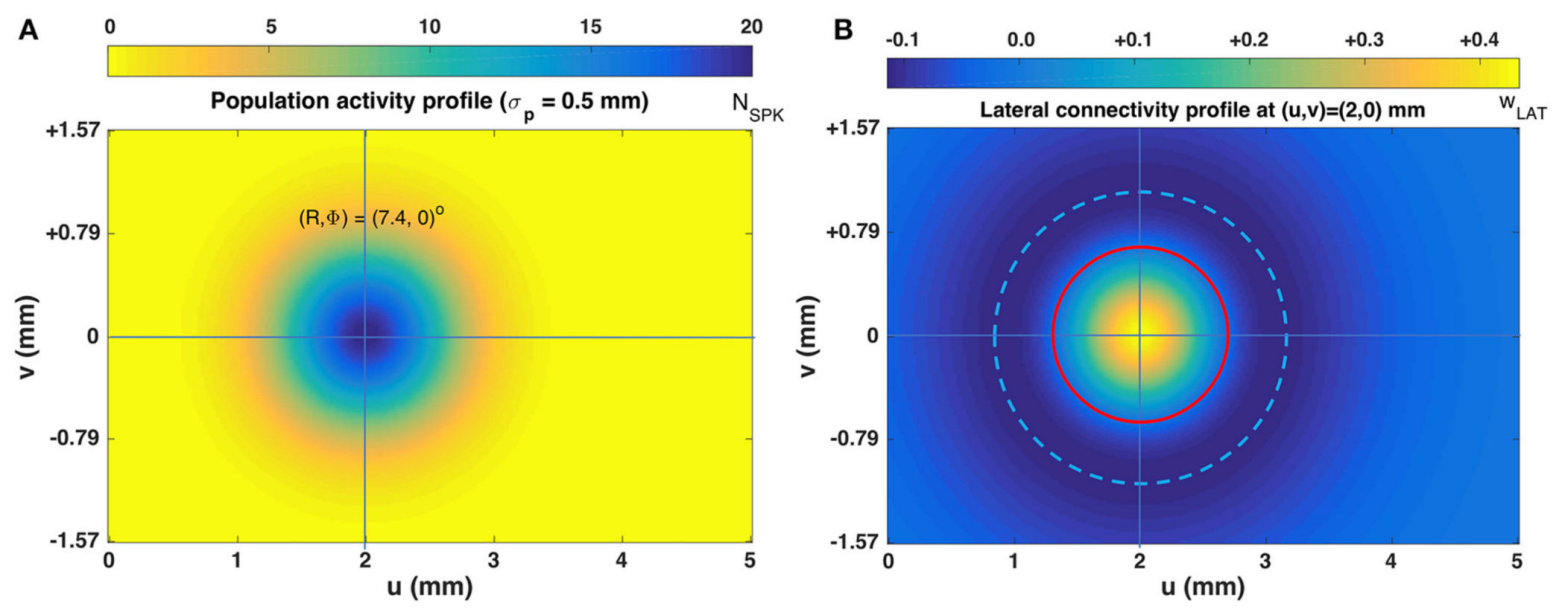
(**A**) Population activity profile for a horizontal saccade with an amplitude of 7.4 deg. The cell in the center of the Gaussian population fires 20 spikes and is located at (u_0_,v_0_) = (2,0) mm (cross hair); the population width is 0.5mm (Equations 2 and 4). (**B**) Excitatory-inhibitory lateral connectivity (in pS) for the cell in the center of the population, according to Equations 12–14, and Table 1. The strongest lateral inhibition is exerted at about 1.1 mm from the cell (light-blue dashed circle). The red circle indicates the w = 0 pS contour, at about 0.6 mm from the cell.

Figure 3B illustrates the lateral connectivity profile for one of the cells [at (*u*,*v*) = (2.0, 0.0) mm] in the motor map, together with the Gaussian population activity around that cell, associated with a small horizontal V-saccade of [R,*Φ*] = [7.4, 0] deg (Figure 2A). Note that the lateral interaction profiles are similar in shape and extent across all cells in the motor map, but the absolute values of the excitatory peak and inhibitory trough decrease in a systematic way with the rostral-caudal coordinate, *u*, as s_(0)_ = 0.0148 and s_(5)_ = 0.0113, from Equation (14).

## Results

### Single-Site Stimulation

**Figure 4:**
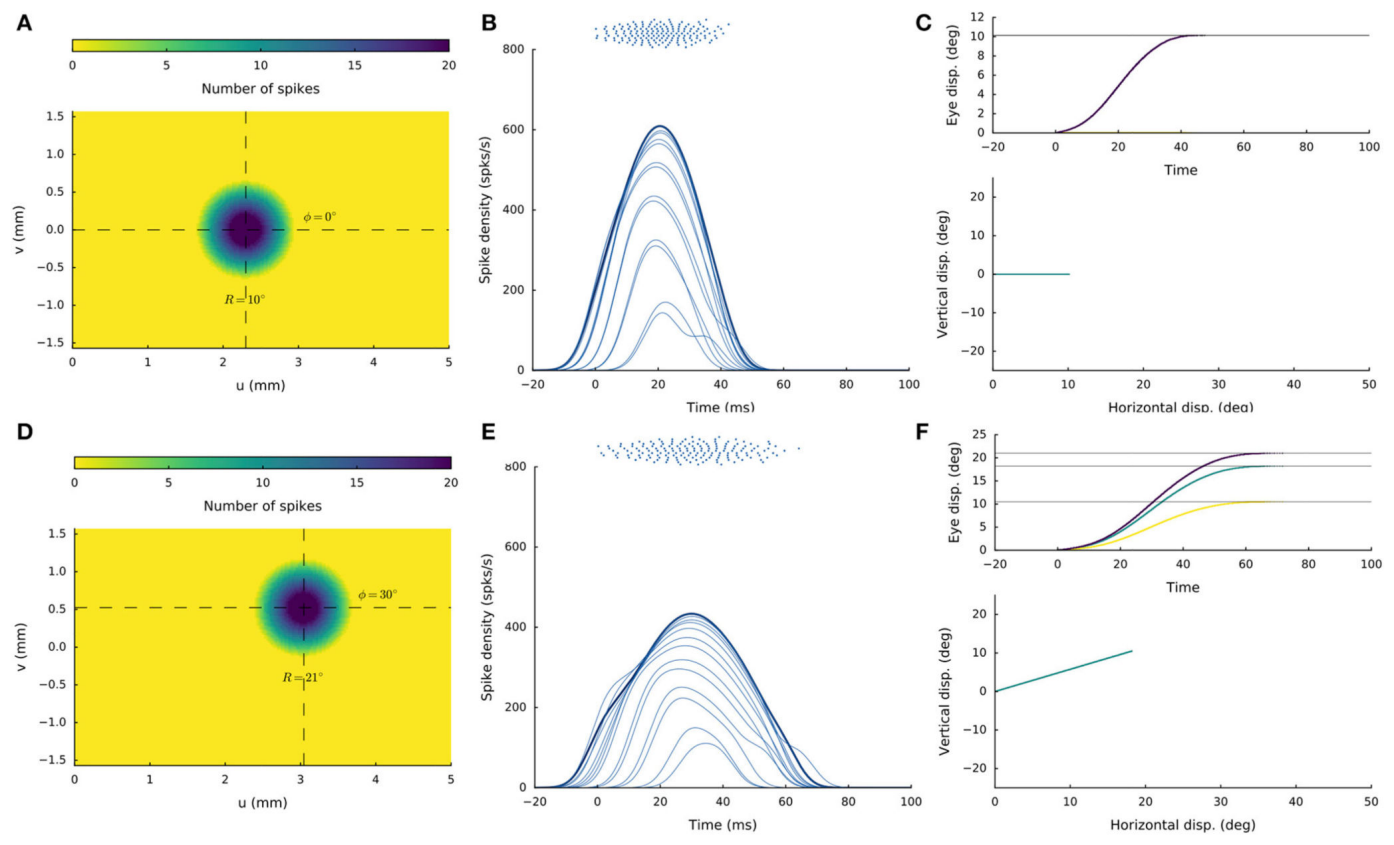
(**A,D**) Cumulative spike counts in the gaze-motor map in response to microstimulation at two single sites. (**B,E**) Temporal burst profiles of the recruited neurons at 0.1 mm intervals from the central neuron illustrate synchronized population activity. Peak firing rates of the cells decrease with distance from the population center, which coincides with the location of the stimulation electrode. Burst durations increase for the larger saccade, but the total number of spikes in both populations remains the same. (**C,F**) Top: Eye-displacement temporal profiles, generated by the linear dynamic ensemble-codg model (Equation 3). Horizontal (green), vertical (yellow), and vectorial (purple) eye-displacement traces. Note the longer duration of the larger movement (main-sequence property), and synchronized horizontal/vertical movement components (stretching). Bottom: 2D straight saccade trajectories.

Figures 4A–C shows the recruited neural population at a rostral stimulation site (R = 2 deg, Φ = 0 deg) for stimulation with an amplitude of *I*_0_ = 150 pA and duration *D_S_* = 100ms. The diameter of the circular population extends to about 1mm in the motor map, with the cumulative spike count of the central cells reaching ~20 spikes. Figure 4B provides the neuronal bursts (top spike patterns) from 12 selected cells, together with their calculated spike-density functions. The peak firing rate of the central cells was close to 700 spikes/s and dropped in a regular fashion with distance from the population center. Note also that the cells near the edge of the population were recruited slightly later than the central cells, but that their peak firing rates were reached nearly simultaneously. Moreover, the bursts all appeared to have the same shape. Figure 4C presents the saccade of 2 deg (top: as function of time; bottom: as a spatial trajectory) encoded by this population through Equation (3).

Figures 4D–F shows the results for stimulation at a more caudal location in the motor map, yielding an oblique saccade with R = 21 deg, Φ = 30 deg. The size of the evoked population activity is very similar to that of the rostral population, and also the number of spikes elicited by the cells is the same. The peak firing rates of the neurons, however, were markedly lower at the caudal site, reaching a maximum of about 450 spikes/s. As a result, the burst durations increased accordingly, from about 35ms at the rostral site, to more than 70ms at the caudal site. Note also that the horizontal and vertical position and velocity temporal profiles are scaled versions of each other, leading to a straight oblique saccade trajectory (Figure 4F, lower panel).

### Synchronous Stimulation at Nearby Rostral-Caudal Sites

**Figure 5:**
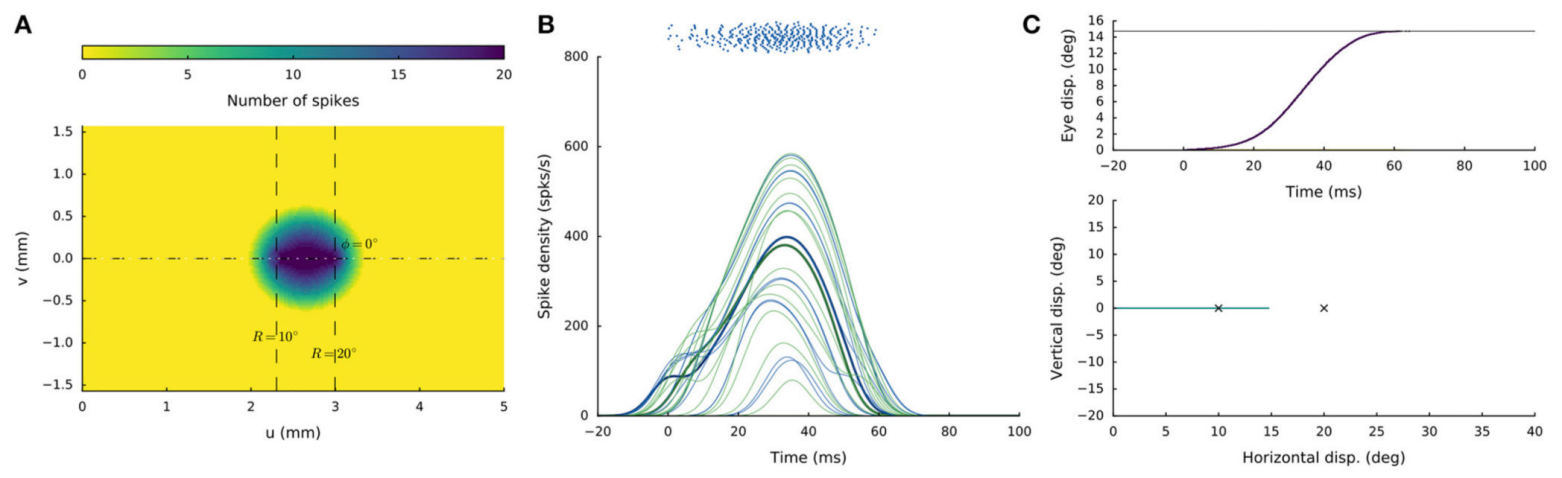
Synchronous double stimulation with the same current strengths (I_0_ = 150 pA) at two nearby sites on the horizontal meridian, corresponding to R = 10 deg (at u = 2.3 mm) and R = 20 deg (at u = 3.0 mm), respectively. (**A**) The neural interactions produce a single population with its peak activity between the two sites. (**B**) Temporal burst profiles of a set of neurons belonging to the active population. The two neurons closest to the stimulation sites reach similar peak firing rates (highlighted profiles). (**C**) The resulting saccade (Equation 3) has an amplitude of 15 deg, which is at the weighted averaged position.

Figure 5 shows the network response to synchronous double stimulation for two nearby sites, at *R* = 10 and R = 20 deg (i.e., *u* = 2.3 and 3.0 mm; Equation 5a) on the horizontal meridian [i.e., *Φ* = 0 (*v* = 0 mm), for both sites]. The microstimulation parameters were taken the same at both locations (*I*_0_ = 150 pA for *D_S_* = 100 ms*)*. After about 30 ms following population activity onset, the highest merged population activity is observed, in which the most active neurons are found between the two stimulation sites (Figures 5A,B). The firing rates of the two neurons closest to the stimulation electrodes are highlighted in Figure 5B. Note that the resulting firing rates at these stimulation sites are markedly lower than at the center of the total population. Note also that these firing rates are highly similar. For single-site stimulation, these firing rates would have been different, due to the tuning properties of the neurons within the motor map (Equation 13). These interesting equilibrating population dynamics result from the mutual excitatory/inhibitory interactions among the neurons, as given by Equations (12, 14) (cf. with Figure 3B).

### Synchronous Stimulation at Widely Separated Rostral-Caudal Sites

**Figure 6:**
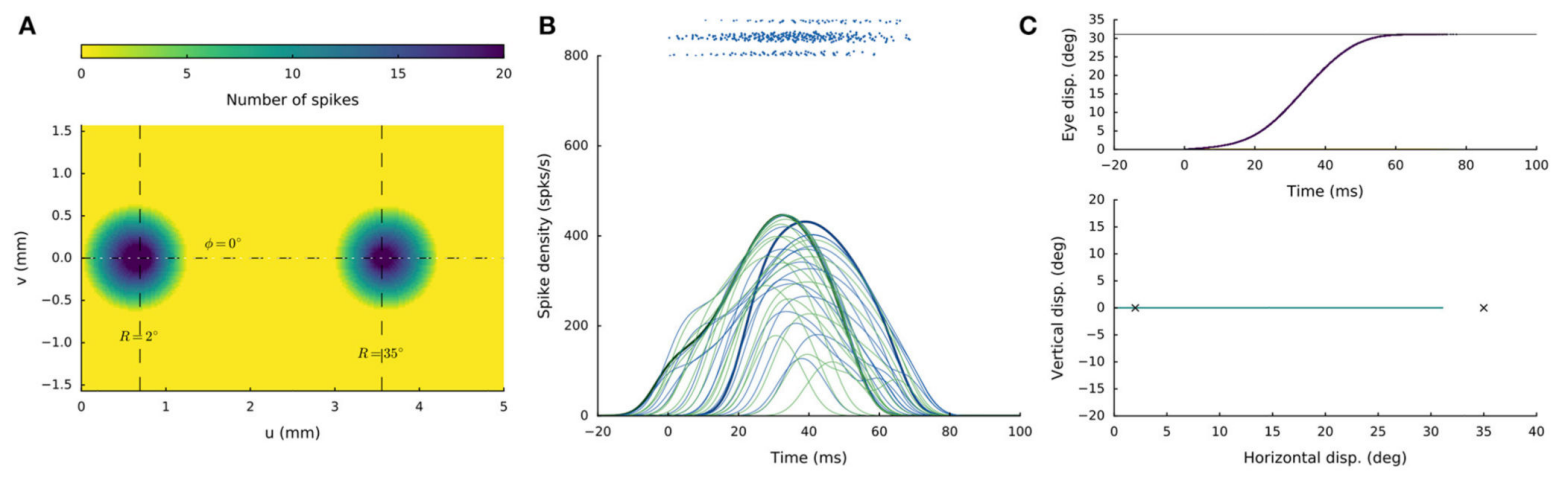
Synchronous double stimulation with the same current strengths at two separated sites on the horizontal meridian, corresponding to R = 2 deg (at u = 0.7 mm) and R = 35 deg (at u = 3.6 mm), respectively. Now, the two stimuli generate two separate populations that together produce a saccade of R = 31 deg. Note that the peak firing rates and burst durations in both populations are similar, but differ markedly from the single-site stimulation rates (cf. with Figure 4).

Figure 6 illustrates the network response to synchronous double stimulation with the same intensity and duration as in Figure 5, at two sites on the horizontal meridian that are separated by nearly 3 mm: *R* = 2 deg and R = 35 deg, respectively (at u = 0.7 and 3.6 mm). About 30 ms after activity onset, two separated populations can be observed, in which the most active neurons now coincide with the two stimulation sites (Figure 6A). The firing rates of the two neurons closest to the stimulation electrodes are again highlighted in Figure 6B. Note that the peak firing rate at the small-amplitude stimulation site (green line) is markedly lower (by almost 50%) and has a much longer duration than for the single-site stimulation result (cf. Figure 4B). Both populations appear to result in comparable firing dynamics, which again is due to the mutual interactions among the neurons across the motor map (cf. with Figure 3B). However, because the strength of the interaction profiles is site-specific (Equations 12-14), the populations show different onset dynamics, with the caudal site starting later than the rostral site.

The resulting horizontal saccade has an amplitude of 31 deg, which differs from the linear summation of the two stimulation effects (R_SUM_ = 37 deg).

### Weighted Averaging for Rostral-Caudal Sites

**Figure 7:**
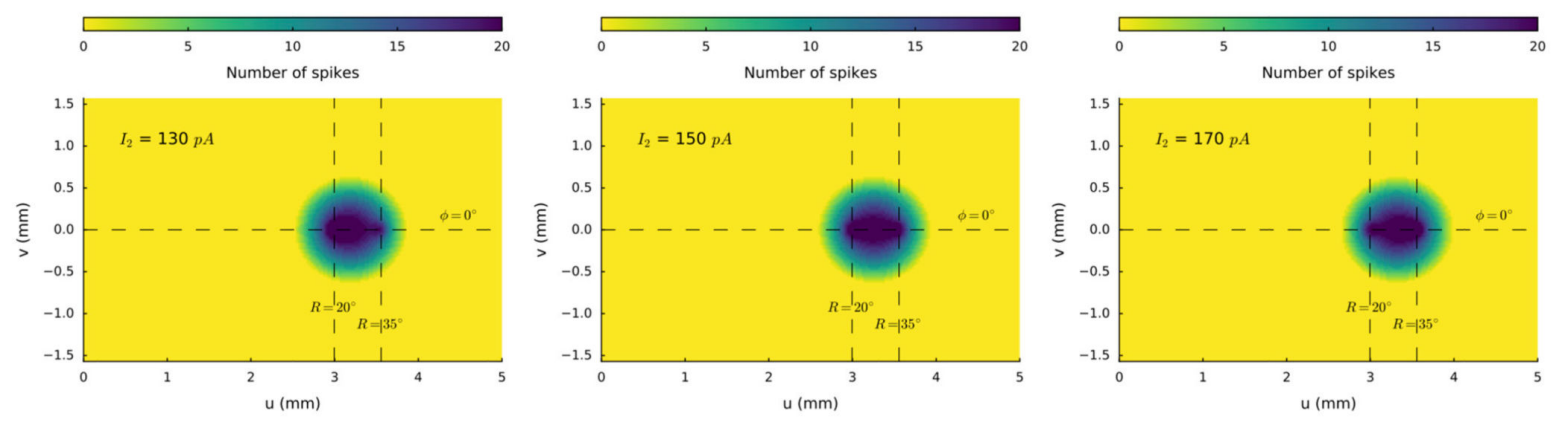
Spike counts of the activated neural populations when the input current at the caudal stimulation site at R = 35 deg is varied from I_0,2_ = 130, 150 and 170 pA, with the stimulus strength at the rostral site (R = 20 deg) kept fixed at I_0,1_ = 150 pA. Note that the center-of-gravity of the merged population shifts in the direction of the stronger stimulation site.

We next illustrate the effect of varying the relative current strengths at two stimulation sites on the horizontal meridian (at R = 20 deg and R = 35 deg, respectively) for synchronous double stimulation. The stimulation amplitude at the rostral electrode was kept constant at *I*_0,1_ = 150 pA, whereas the stimulus intensity at the caudal site was varied systematically between *I*_0,2_ = 100 and 200 pA in 10 pA steps. Figure 7 illustrates three stimulus situations: *I*_0,2_ = 130 pA, *I*_0,2_ = 150 pA, and *I*_0,2_ = 170 pA. In all three cases a merged population is seen, in which the center-of-gravity of the activity gradually shifts from the rostral to the more caudal site.

**Figure 8:**
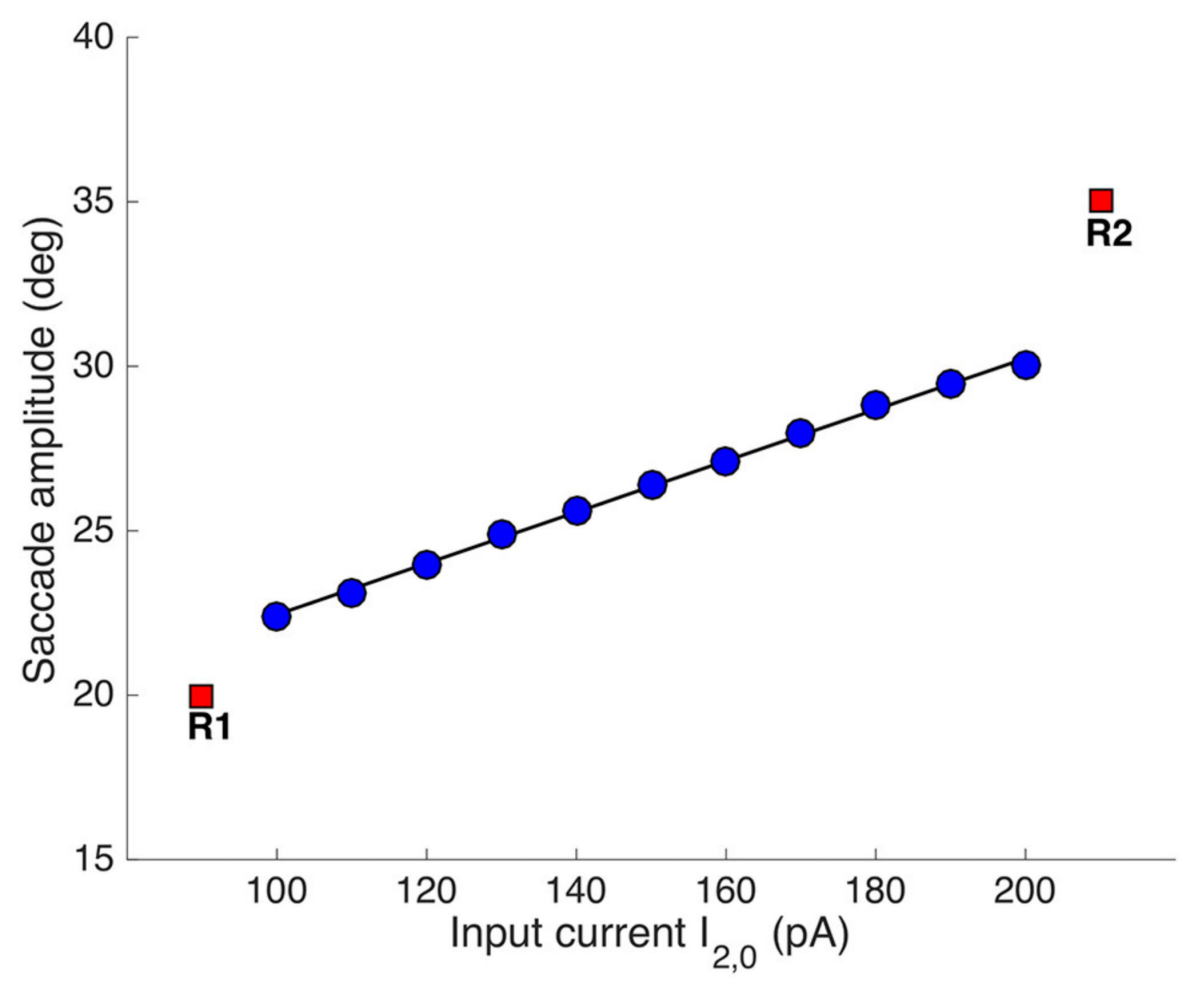
Synchronous double stimulation with varying current strengths at the caudal stimulation site. The input current at [R,Φ] = [35, 0] deg varied between 100-200 pA, while it was fixed to 150 pA at [R,Φ] = [20, 0] deg (same stimulus durations of 100 ms). Varying the stimulation strengths shifted the merged population’s center-of-gravity as in Figure 6. The resulting eye-displacement vectors varied from 22.4 to 30 deg (slope of the linear regression line: 7.8 deg/100 pA).

Figure 8 shows the result of systematically varying the relative stimulus intensities on the evoked saccade amplitudes (all saccades were horizontal, like in Figures 4, 5). The individual stimulation sites produced saccades of R = 20 and R = 35 deg, respectively (red symbols). Synchronous stimulation at the two sites, with *I*_1,0_ = *150 pA* (fixed), resulted in eye-movements with amplitudes that systematically varied as a function of *I*_2,0_ between 22.4 and 30 deg.

### Double Stimulation at Medial-Lateral Sites

**Figure 9:**
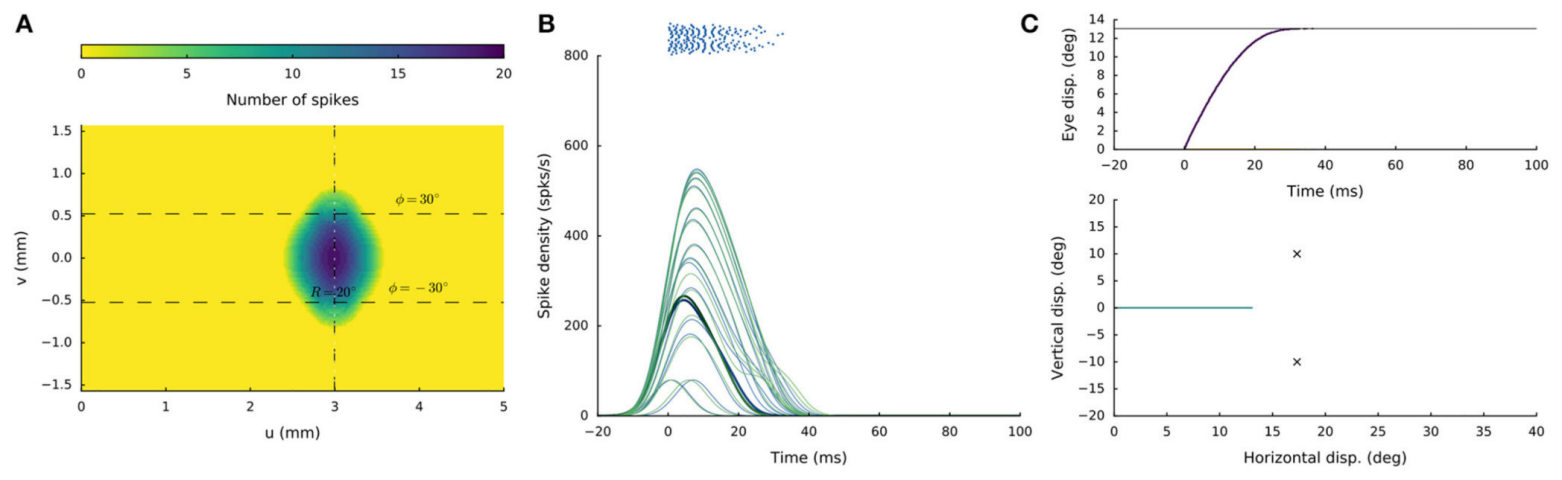
Synchronous double stimulation at the same current strengths at two separated sites, corresponding to [R,Φ] = [20,+30] deg, and [R,Φ] = [20,−30] deg respectively. The two stimuli yield a merged population, and a saccade of R = 13 deg, which is directed toward an average location of the two individual stimulation effects.

We next illustrate the effects of synchronous stimulation at two sites that encode the same saccade amplitude (*u* = constant), but different saccade directions (different *v* coordinates). In Figure 9 the two stimulation electroes were placed at R = 20 deg and were separated by ΔΦ = 60 deg around the horizontal meridian (cf. Figure 2A). The resulting activity shows a merged population with its most intensely firing cells located on the horizontal meridian at R = 20 deg (*u* = 3 mm). In Figure 9B we show the SC bursts for a group of selected cells, with the two sites corresponding to the up and down electrode highlighted by the bold green and blue lines, respectively. Note that the stimulation sites are markedly less active than the cells near the horizontal meridian, and also that their firing rates are much reduced (by more than 40%) with respect to the single-site stimulation effect (cf. Figure 4D). The sites near the horizontal meridian, on the other hand, display firing rates (>500 spikes/s) that significantly *exceed* the peak firing rate (~450 spikes/s) of the single-site stimulation effect at the coordinate for a comparable saccade amplitude.

The resulting saccade is horizontal and has an amplitude of R = 13 deg. In other words, the amplitude is much smaller than the saccade corresponding to the site of maximal activity, which would be R = 20 deg. It is also somewhat smaller than the projection of the saccade vectors onto the horizontal meridian, which would correspond to an amplitude of R_CoG_ = 20·cos(30) = 17.3 deg (cf. Figure 2C).

### Double Stimulation: Evoked Saccade Amplitude Depends on Medial-Lateral Separation

**Figure 10:**
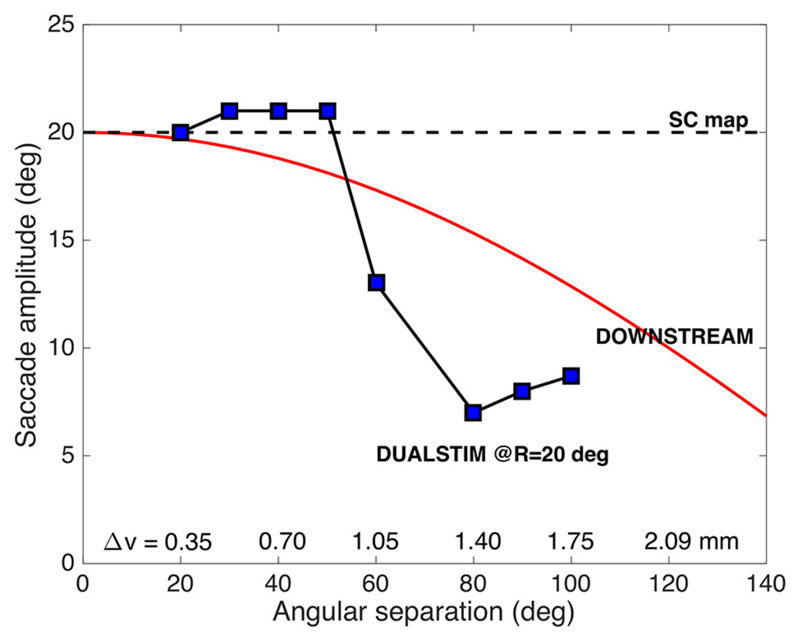
Saccade amplitude as function of electrode angular separation ΔΦ for medial-lateral sites (separated by Δv mm) along the fixed R = 20 deg radius (u = 3.0 mm). Note that the stimulation-evoked saccade amplitudes strongly depend on the medial-lateral distance, and that they vary in a very different way than predicted from center-of-gravity computations (cf. Figure 2C; Equation 4).

To appreciate the complex interactions between the neural populations along the medial-lateral (*v*) axis in the motor map, Figure 10 shows the results for the evoked saccade amplitude (blue symbols) as function of the medial-lateral separation, Δ*v*, or, equivalently, as function of the angular separation between the two single-site movements. The figure also indicates the simple predictions from the pure center-of-gravity calculations that would result from the motor map (R = 20 deg for all sites), and from downstream averaging (the red line). It is clear that the evoked saccades follow neither prediction. Although the averaging effects are clearly due to the neural interactions with the SC motor map (as we have not incorporated a downstream center-of-gravity mechanism in our model, see Equation 3), they clearly differ from the simple scheme of center-of-gravity computation. Instead, the results reflect the intricate neural dynamics as well as the influence of the lateral excitatory-inhibitory interactions (see Figure 3B).

For example, for small spatial separations (up to about 0.7mm), the two populations strongly overlap (as in Figure 9). As a result, they are partly dominated by the mutual excitatory interactions, leading to a slight increase in the saccade amplitude by about one deg. When the sites are separated by about 1mm, both populations undergo mostly inhibitory influences, leading to a reduced saccade amplitude. This effect increases up to about Δ*v* = 1.4 mm, where the evoked saccade (at these current levels) reaches a minimum of 7.0 deg. In this region the inhibitory interactions are the strongest (see Figure 3B). As the electrodes are positioned further apart, the saccade amplitude is still small, but slightly increases up to about 9 deg, because of the slightly lower strength of the lateral inhibition.

### Lateral-Medial Double Stimulation at Different Current Strengths

**Figure 11:**
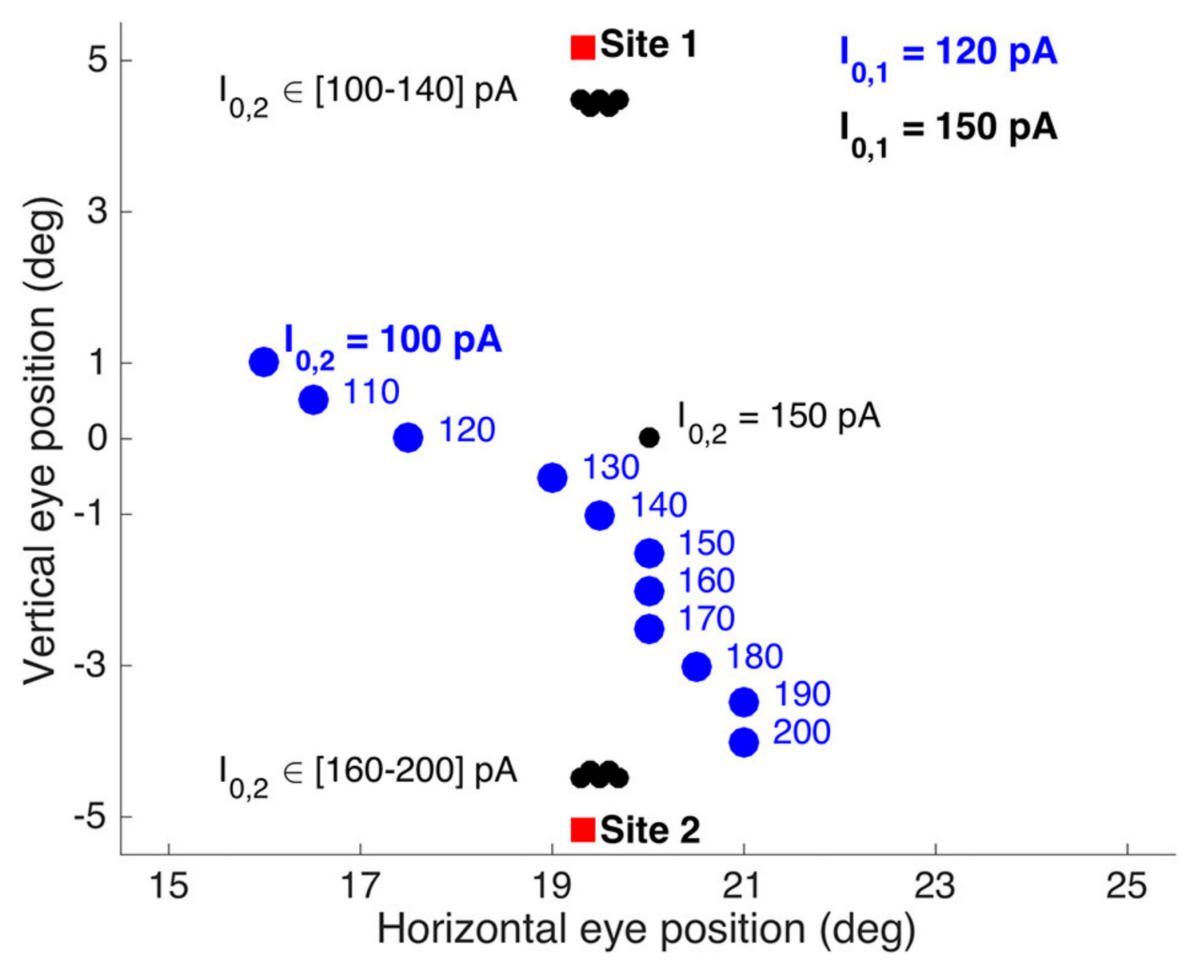
Different double-stimulation response behaviors for the conditions in which the electrode at site 1 (at (R,Φ) = (20,15) deg) was kept fixed and slightly above the saccade threshold at I_0_,1 = 120 pA (blue symbols), or well above the threshold at I_0,1_ = 150 pA (black symbols), while the current at site 2 (at (R,Φ) = (20,−15) deg) was varied from I_0,2_ = 100 to 200 pA in 10 pA steps. The former condition (blue) yielded clear weighted averaging between the effects from the two sites, while the latter condition (black) shows bistable response behavior. Red symbols: single-site evoked saccades at I_0_ = 150 pA.

Weighted saccade averaging can also occur when the electrodes are positioned along the medial-lateral axis, but the effects resulted to depend strongly on both the electrode separation and on the strengths of the two currents. For example, when one electrode was kept fixed at the supra-threshold stimulation intensity of *I*_0,1_ = 150 pA, and the other electrode was varied between *I*_0,2_ = 100–200 pA, the following pattern emerged for all angular separation conditions:

(i) For currents below *I*_0,2_ = 150 pA, site 1 always fully dominated, and all saccades were directed toward the first site.
(ii) Above *I*_0,2_ = 150 pA, site 2 dominated and saccades were directed to the second site.
(iii) Only when the currents were equal, *I*_0,1_ = *I*_0,2_ = 150 pA, averaging was obtained according to the relationship seen in Figure 9. In other words, in these double-stimulation conditions the saccade direction behaved as a bistable variable. This response behavior is illustrated in Figure 11 for an angular separation of 30 deg (Δ*v* = 0.52 mm; black symbols).

True averaging of the saccade direction was only obtained when (i) the fixed stimulation current at site 1 was lowered to slightly above the threshold for evoking a saccade (e.g., to *I*_0,1_ = 120 pA), and (ii) the two sites were close together. Figure 11 shows the results of such weighted stimulation effects for the same sites (blue symbols). The figure shows that from *I*_0,2_ = 130 pA onwards, a clear weighted averaging pattern was obtained, in which the saccade direction varied systematically with the difference in current strength. Note that for currents below about *I*_0,2_ = 130 pA, also the saccade amplitude started to decrease, as for these cases both currents were getting close to their saccade-evoking thresholds.

### Double Stimulation With Delay

**Figure 12:**
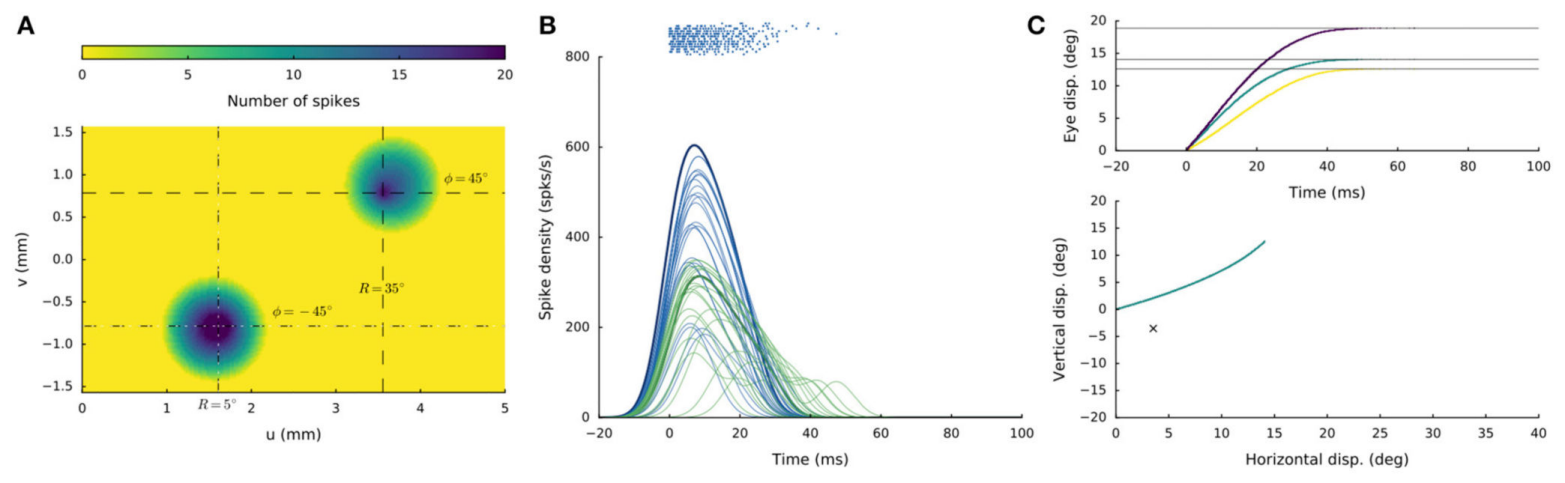
Supra-threshold (150 pA) double stimulation with a short inter-current delay. (**A**) Spike counts of the active populations at stimulation sites [R_1_,Φ_1_] = [5, −45] deg and at [R_2_,Φ_2_] = [35,+45] deg, when the input current at the latter site was delayed by 2 ms. (**B**) Firing rates of the cells in the active populations are plotted in different colors (blue and green for the first and second population, respectively). (**C**) Resulting eye-displacement components as function of time (top) and the 2D eye-movement trajectory (bottom). Note that the saccade trajectory is curved, as the initial and final directions of the movement are different.

In a similar way as observed for the interactions along the medial-lateral coordinate (see sections Double Stimulation: Evoked Saccade Amplitude Depends on Medial-Lateral Separation and Lateral-Medial Double Stimulation at Different Current Strengths), imposing a temporal delay between the two supra-threshold electrode currents (when both at 150 pA) produced different response behaviors, depending on the electrode separations and current strengths. For supra-threshold stimulation at both sites, a curved saccade trajectory would only emerge when the delay was very short (typically, below 6ms), and the stimulation sites are separated in both the medial-lateral and rostral-caudal dimensions of the motor map. An example of such a stimulation condition is shown in Figure 12. The two sites were at [R,Φ] = [5,−45] and [35,+45] deg, respectively, and the current strengths were 150 pA at both sites, whereby the stimulation pulse at the second site was delayed by 2ms. Both electrodes set up a population response, leading to a curved saccade trajectory with an overall amplitude of R = 19 deg and a direction of about Φ = 40 deg, which is a weighted average of the individual stimulus effects. When the delay was increased to 4ms the initial direction of the saccade was horizontal curving toward the final site location in midflight of the response (not shown).

At delays above 5ms, the saccade was invariably directed at the endpoint of the first site, as the second site would be strongly inhibited by the activated first population. As a result, the second site would not be able to set up an appropriate population response to produce a colliding saccadic on its own.

When the stimulation sites and current strengths, as well as the delays were systematically varied, the occurrence of curved saccade trajectories resulted to be quite rare. Instead, we often obtained a bistable response behavior, in which a small change in one of the stimulation parameters (e.g., the current strength at the first electrode) could fully change the saccadic response from being directed to the first site, toward the second site.

**Figure 13:**
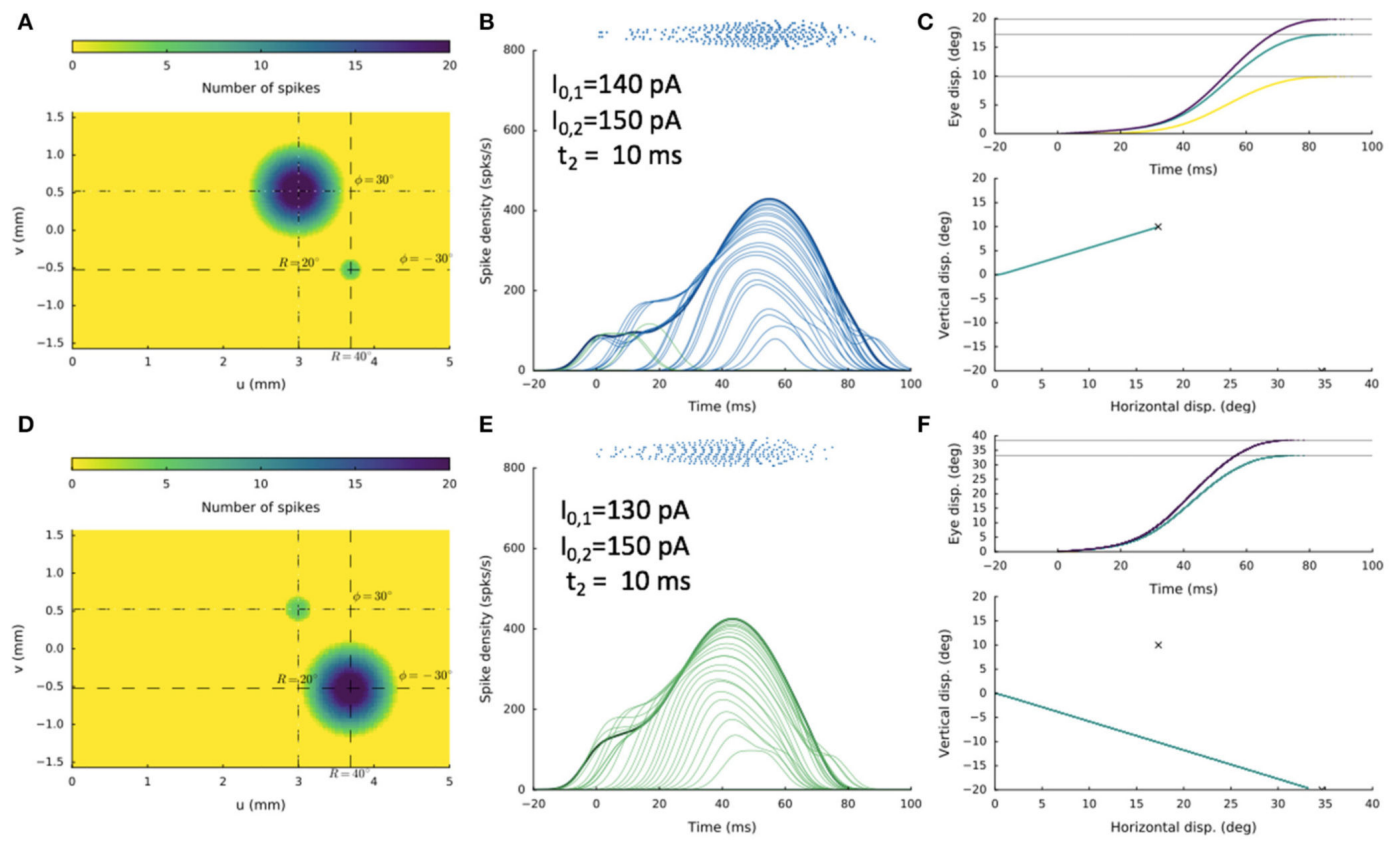
Double stimulation with a 10 ms delay, for two sites about 1.3 mm apart, showing high sensitivity of the network to small changes in the stimulation parameters. In (**A–C**) the current at the first electrode was I_0,1_ = 140 pA, whereas in (**D–F**) it was only slightly lowered to I_0,1_ = 130 pA. Yet, the resulting saccades differed dramatically, in line with bistable response behavior.

An example of this bistable behavior on the stimulation conditions is shown in Figure 13, where the two sites were at [R_1_,Φ_1_] = [20,+30] deg and [R_2_,Φ_2_] = [40,−30] deg, respectively, and the delay was 10 ms. The stimulation current, I_0,2_, was 150 pA in both cases, whereas I_0,1_ was either 140 pA, or 130 pA. In the former condition, a straight saccade is directed toward site 1, whereas in the latter case, a straight saccade is made in the direction of site 2.

We systematically varied the inter-stimulus delay *t*_2_ from (2, 5, 10, 20, 50) ms and *I*_0,1_ from (200, 190, …, 80) pA (*I*_0,2_ fixed at 150 pA), and obtained similar bistable results for many cases. Note, however, that these two sites are separated by about 1.26mm, which falls in the strongest inhibitory range of the lateral connectivity profile. In the situation of Figure 12 the two sites are further apart, given weaker mutual inhibition and allowing more excitatory interactions (see Figure 3B and section Discussion).

## Discussion

### Summary

Synchronous double stimulation in a spiking neural network model of the SC with Gaussian excitatory-inhibitory interactions results in saccade responses that display many of the features that have been reported in electrophysiological studies [9, 25, 34]: when the electrodes were located on an iso-direction line (*v* = constant) the resulting saccade amplitudes were a weighted average of the individual stimulus effects, with the current strengths acting as weighting parameters (Figures 5–8). When the electrodes were positioned along iso-eccentricity lines (*u* = constant), however, the response patterns appeared to be more complex: weighted averaging was obtained for low stimulation currents at nearby stimulation sites, but when the electrodes were moved further apart and/or the current levels increased, we obtained bistable response behavior (Figures 9–11). When a delay was introduced between the first and second stimulus pulse, the averaged saccade trajectories could become curved, provided the delay was short (<6 ms; Figure 12). For longer delays, saccades were invariably directed toward the site evoked by the first electrode when its current intensity was above the normal saccade-initiation threshold (150 pA). In other cases, we obtained bistable response behavior, in which the saccade was directed either to the first site, or to the second site, without averaging (Figure 13).

The weighted averaging effects, which betray a nonlinearity in the system, are entirely due to the neural dynamics (Equations 6–7) and synaptic connectivity patterns (Equations 12–14) within the SC motor map, as the downstream motor circuitry in our model was taken entirely linear (Equation 3). Yet, the averaging results of our simulations do not correspond at all to the simple prediction of a center of gravity calculation at the level of the motor map either (Equation 4a; Figure 2B), as for iso-eccentricity stimulation the evoked saccade amplitudes varied strongly with the electrode separation (Figure 10), in a pattern that somewhat resemble the effect of downstream averaging. Whether these predictions truly deviate from observed experimental data on synchronous double stimulation is hard to tell, as precise measurements and quantification of this phenomenon are rare (e.g., 25, 34). The same may hold for the exact paths followed by curved trajectories evoked by delayed electrical double stimulation [25, 34, 39].

In what follows, we discuss these apparent discrepancies with the experimental data.

### Model Structure

The subtle different behaviors observed for iso-direction vs. iso-eccentricity stimulation are likely caused by the differences in neural organization for the *u*- and *v*-coordinates in our model. The tuning parameters of the neuronal dynamics (the adaptive time constant, Equation 13) and the lateral synaptic projection strengths (the scaling parameter, Equation 14) both only vary with the rostral-caudal coordinate (*u*), and are assumed constant along iso-eccentricity lines.

These biophysical neural tunings were required to explain the firing behavior of collicular neurons under single-site visual stimulation conditions [8, 13, 23], and the nonlinear saccadic main sequence kinematics (see Introduction). From our single-unit recordings we noted that the peak firing rates of SC neurons in the center of the population decreased systematically with the saccade amplitude, meanwhile increasing their burst durations to keep the number of spikes in the saccade-related burst invariant across the motor map for slow, fast, small and large saccades. As single-site microstimulation produces normal saccadic eye movements, we argued that the same population activity would emerge during electrical stimulation and for natural visual stimulation. The neural population dynamics are then explained by synaptic lateral interactions, and are hardly influenced by the externally applied electrical stimulation current. We assumed that the stimulation current directly activates only a small subset of the neurons around the electrode. Indeed, under these assumptions, most single-site microstimulation results could be accounted for as well [26].

One discrepancy with experimental observations concerned the near-threshold behavior of the network: around the stimulation threshold, the network’s saccades become much slower than main sequence (as evoked firing rates decrease), but their size (determined by the total number of spikes in the burst) remained unaffected. However, experiments have revealed that near the threshold, saccades become both slower than main sequence *and* smaller [15, 35]. This would suggest that near threshold not only the firing rates are reduced, but also the number of spikes. The current model does not incorporate this possibility.

We here conjecture that the failure to produce different numbers of spikes for near-threshold conditions may also underlie the bistable character of our model to some of the double-stimulation conditions, and its reluctance to readily produce curved saccades. In double stimulation, the two electrodes exert a mutual inhibitory influence, which brings the weaker stimulation site to near- or below-threshold levels under many conditions. Indeed, when the stimulation sites fall in each other’s strongest inhibitory zones, the bistable effects are nearly impossible to overcome (e.g., Figures 11, 13). On the other hand, when the stimulation electrodes are placed along the *u*-direction in the map, bi-stability is less common. This is probably due to the decreasing strength of the lateral connectivity patterns along this dimension, as dictated by Equation 14 (the most caudal sites exert nearly 25% less influence than the most rostral sites).

One possibility to overcome this discrepancy is to introduce variability (noise) in the neural population, e.g., at the level of the synaptic conductances (Equation 11), and at the adaptive time constants (Equation 13), that relies on the total input strength to the neuron (multiplicative noise; [8]). This will affect the total number of spikes of the neuron, and therefore could potentially lead to smaller saccades for effectively weak inputs.

### Untested Predictions

The neural interactions, imposed by the two separated electrodes, cause some interesting (and somewhat unexpected) behaviors of the neural firing properties, which so far have not been tested experimentally. Under single-site stimulation, the activity of the central cell, which encodes the ensuing saccade amplitude and direction, fully determines the firing-rate profile of all other cells, as well as the saccade kinematics (neural synchronization; e.g., Figure 4). Under double-stimulation at different nearby sites, however, the most active cells are no longer found at the stimulation electrodes, but at a location in between. The firing rates of these most active cells now determine the full saccade kinematics and the firing profiles of the other cells (e.g., Figures 6, 7, 9). Interestingly, the kinematics of the resulting saccades (which are slower) and the firing rates of these most active cells (which are higher) differ from the effects of single stimulation at that most active site. Unfortunately, it is difficult to test this prediction experimentally for the firing rates under electrical double stimulation, because of the strong electrical artifacts produced by the electrodes.

However, the effects of double stimulation on the emerging eye-movement kinematics can be readily assessed. As far as the main-sequence properties are concerned, averaging saccades under double visual stimulation appear to be slower than saccades of the same amplitude to a single visual stimulus, and the associated firing rates in the SC are lower (e.g., [46]). To our knowledge, the detailed velocity profiles under electrical double-stimulation have so far not been quantified in experimental studies.

### Lateral Interactions

The simulations of electrical double stimulation made clear that the shape of the Mexican-hat profile affects the activity profiles of both active neuron populations and of the resulting saccades (e.g., Figure 11). The presence of lateral interactions within the SC has been well established by both anatomical and physiological evidence [28, 30, 33]. Modeling studies have suggested different synaptic interaction profiles, such as local excitation and global constant inhibition [37], or Mexican-hat type Gaussian profiles [45]. In the present study, we fixed the ranges of the excitatory and inhibitory interactions (*σ_exc_* and *σ_inh_*) for all cells and tuned their synaptic strengths in line with the proposal of Trappenberg et al. ([45]; Equation 14). Although it is conceivable that different profiles with shorter ranges could generate similar population activities (see below), anatomical studies so far do not allow to quantify the connectivity profiles and ranges, except for recent *in-vitro* studies [31, 32].

In contrast to the model of Van Opstal and Van Gisbergen [38], in the present model the effective range of the electrical current was assumed to be small (Equation 10; [26]). This assumption was inspired by recent findings from stimulation experiments with simultaneous calcium imaging in frontal cortical tissue [27, 47]. In our model, the stimulation profile is subsequently combined with the Mexican-hat interaction function of Equations 12–14. We have shown earlier, using a static population model of the SC, that a weak global constant inhibition in combination with a delta function for the excitatory profile (i.e., only self-excitation) could yield saccade-averaging results if the current-spread function was a Gaussian with a much broader extent as in the present study, and whereby its width depended in a nonlinear way on the applied current strength [38].

Note that for network models such as these, including our own, the overall spatial effect of the stimulation (ignoring time) is in fact given by the convolution of the electrical stimulation profile with the weighting kernel of the excitatory-inhibitory interactions. Each cell’s membrane potential is thus described by:

(15) $${\text{V}}_{\text{n}}\left(\text{u},\text{v}\right)=\int \int_{{\left(\text{u},\text{v}\right)}_{\text{min}}}^{{\left(\text{u},\text{v}\right)}_{\text{max}}}{\text{w}}_{\text{n}}\left(\sigma ,\tau \right)\cdot {\text{I}}_{\text{INP}}\left(\text{u}-\sigma ,\text{v}-\tau \right)\cdot \text{d}\sigma \text{d}\tau$$

which constitutes one equation for the membrane potential of neuron n, as a multiplicative combination of two functions. It is therefore conceivable that many potential functions could fulfill Equation 15. However, the nonlinear dynamics of the current model (Equations 6–7) makes a simple analytical approach to find the optimal solution that satisfies all experimental constraints not feasible. Further study is therefore required to analyze the effects of different profiles on the total network behavior across a wide range of sensory and electrical stimulation conditions.

As a final note, the electrical stimulation inputs were simply taken as constant rectangular pulses, instead of trains of short-duration stimulation pulses. In the latter case, which is physiologically more realistic, also the pulse intervals (stimulation frequency), pulse durations (stimulus train lengths), pulse heights, pulse interleave times, and pulse polarity may all play a role in the evoked E-saccades under single and double stimulation paradigms [24, 25, 34]. Incorporating these different stimulation parameter settings in our spiking neural-network model will require some tedious retuning of the network parameters, but may be worth the effort for its potential to generate novel neural dynamics.

## Figures and Tables

**Table 1:** List of all parameters used in the simulations.

**MICROSTIMULATION PARAMETERS**		

*λ*	10 mm^−1^	Spatial decay constant
*I*_0_	150 (40−280) pA	Intracellular current intensity
*P*(*t*)	*I*_0_ (for 0 < t < D_S_)	Rectangular stimulus pulse
*D_S_*	100 (25 - 250) ms	Stimulation duration

**NEURAL PARAMETERS**		

*C*	600 pF	Membrane capacitance
*g_L_*	20 nS	Leak conductance
*E_L_*	−53 mV	Leak reversal potential
*η*	2 mV	Spike slope factor
*V_T_*	−50 mV	Exponential threshold
*V_peak_*	−30 mV	Spiking threshold
*V_rst_*	−45 mV	Reset potential
*a*	0 nS	Sub-threshold adaptation
*b*	120 pA	Spike-triggered adaptation
*τ_q_*	100-30 ms	Location-dependent adaptation time constant; varies with (*u_n_*) (Equation 13)
*ζ*	5.087·10^−5^	Spike-vector scaling

**SYNAPTIC PARAMETERS**		

*E_exc_*	0 mV	Excitatory reversal potential
*E_inh_*	−80 mV	Inhibitory reversal potential
*τ_exc_*	5 ms	Excitatory conductance decay
*τ_inh_*	10 ms	Inhibitory conductance decay

**LATERAL CONNECTIVITY PARAMETERS**		

*w_exc_*	45 pS	Excitatory scaling factor
*σ_exc_*	0.4 mm	Range of excitatory synapses
*w_inh_*	14 pS	Inhibitory scaling factor
*σ_inh_*	1.2 mm	Range of inhibitory synapses
*s_n_*	0.0113−0.0148	Location-dependent synaptic scaling parameter; varies with (*u_n_*, Equation 14).
